# Hepatitis B and C Viruses' Infection and Associated Factors among Pregnant Women Attending Antenatal Care in Hospitals in the Amhara National Regional State, Ethiopia

**DOI:** 10.1155/2020/8848561

**Published:** 2020-10-09

**Authors:** Mulat Dagnew, Yihenew Million, Mucheye Gizachew, Setegn Eshetie, Gashaw Yitayew, Lakachew Asrade, Mulat Adefris, Feleke Moges, Moges Tiruneh

**Affiliations:** ^1^University of Gondar, College of Medicine and Health Sciences, School of Biomedical and Laboratory Sciences, Department of Medical Microbiology, Gondar, Ethiopia; ^2^Bahir Dar University, College of Medicine and Health Sciences, School of Medicine, Department of Obstetrics and Gynecology, Bahir Dar, Ethiopia; ^3^University of Gondar, College of Medicine and Health Sciences, School of Medicine, Department of Obstetrics and Gynecology, Gondar, Ethiopia

## Abstract

**Introduction:**

Hepatitis virus infection is a major public health burden and silent killer disease in sub-Saharan Africa, including Ethiopia. Therefore, this study aimed to investigate the prevalence of hepatitis B and C viruses and associated factors among pregnant women attending an antenatal clinic in three tertiary hospitals in Amhara National Regional State, Ethiopia.

**Methods:**

A cross-sectional study was conducted among 1121 pregnant women. Data on sociodemographic and associated factors were collected using a structured questionnaire. Serum samples were tested for hepatitis B surface antigen (HBsAg) and anti-hepatitis C virus antibody (anti-HCV) using ELISA. SPSS version 20 was used for data analysis, and a multivariable logistic regression analysis was used to assess the relationship between factors associated with hepatitis B virus and hepatitis virus C infection.

**Results:**

A total of 1121 pregnant women were included in the study. The mean age of study participants was 27.2 ± 4.8 yrs. The majority of pregnant women (895 (79.8%)) were from urban areas. The overall seroprevalence of HBsAg and anti-HCV antibody was 52 (4.6%) and 18 (1.6%), respectively. The coinfection rate of HBV/HCV was 1.4% (1/69). Ten (19.2%) of HBV positive cases were coinfected with HIV. There were no coinfections of HCV and HIV. Interestingly, pregnant women with a history of multiple sexual partners (AOR = 3.2, 95% CI, 1.7–7.6), blood transfusion (AOR = 7.6, 95% CI, 2.9–16.9), family history of HBV (AOR = 3.5, 95% CI, 1.7–7.6), being HIV-positive (AOR = 2.5, 95% CI, 1–5.9), and tattooing (AOR = 2, 95% CI, 1–3.8) were significant predictors of HBV infection. Similarly, young age (17–25 yrs) (AOR = 3.2, 95% CI, 1.8–8.6) and no educational background (AOR = 5, 95 CI, 1.7–14.8) were significant predictors of HCV infection.

**Conclusions:**

Hepatitis B and C viruses' infection was intermediate among pregnant women; some risk factors were significantly associated with the majority of cases. Infants born from these infected mothers are at risk of infection. This calls for screening and integration of HBV prevention of mother-to-child transmission (PMTCT) into HIV. Thus, the provision of health education on hepatitis B and C viruses' transmission, vaccination, and screening of all pregnant women routinely are essential for the prevention of these viruses.

## 1. Introduction

Viral hepatitis is a major public health burden all over the world. It is responsible for an estimated 1.4 million deaths, which is greater than the death toll of 1.2 million to that of HIV. Globally, 248 million and 150 million people have chronic hepatitis B virus (HBV) and hepatitis C virus (HCV) infections which cause death for 780,000 and 350,000 per year, respectively [1]. HBV and HCV infections are highly endemic in developing regions of Asia and Africa [2, 3]. Despite its high prevalence of HBV and HCV, it remains underreported and lacks reliable epidemiological data in most African countries, including Ethiopia. Globally, less than 5% of persons living with chronic viral hepatitis are aware of their status [1]. The majority of hepatitis infected individuals remain asymptomatic or apparently healthy, transmit the virus to other persons, and die of the infection without notice; it is a silent killer [4].

Hepatitis B virus is highly contagious and a hundred times more infectious than HIV, transmitted horizontally through infected blood, blood products, unprotected sex, unsafe injection, and tattooing and vertically from infected mother to child, before birth, during birth, and after birth [5, 6]. Mother-to-child transmission (MTCT) of HBV remains a major source of chronic infection in endemic countries [7]. A pregnant woman positive for HBsAg, hepatitis B e antigen (HBeAg), and HBV viral load of deoxyribonucleic acid (DNA) (>200,000 IU/mL, equivalent to >5.3 log10 copies/mL), the chance of MTCT increases and reaches 90% [8]. In Africa, about 70–90% of infants, infected before 1 year of age, develop chronic HBV infection, liver cirrhosis, hepatocellular carcinoma, and early death in children [9]. To prevent MTCT, screening, early case detection, initiation of treatment of pregnant women, and provision of active birth dose HBV and passive hepatitis B immunoglobulin (HBIG) vaccines within 24 hours are widely recommended for infants born to HBsAg-positive pregnant mothers [9, 10].

Hepatitis C virus is transmitted mainly through parenteral routes such as infected blood transfusion, intravenous drug use or blood products, therapeutic injection, intravenous drug use, acupuncture, tattooing, ear-piercing, and transmission during sexual contact and vertically from mother to child [5, 11, 12]. Vertical transmission of HCV from mother to child contributes to 10% of cases and is lower than other viral pathogens such as HIV and HBV [13]. Drugs like direct-acting antiviral agents (DAAs) are effective for curing up to 70% of HCV-infected persons; however, they are contraindicated during pregnancy [11, 13]. Currently, there is no effective vaccine for HCV and no effective means of preventing MTCT of HCV.

Viral hepatitis during pregnancy is associated with a high risk of maternal, fetal, and neonatal complications such as stillbirth, abortion, premature birth, and low birth weight [14]. Despite this problem, currently, screening for cancer-causing hepatitis viruses is not a routine practice in health institutions in Ethiopia due to a lack of a program, a lack of diagnostic kits, and high cost of treatment. Accordingly, the World Health Organization (WHO) has set an ambitious plan to reduce 50% MTCT of HBV, less than 0.1% HBsAg in children by 2020, and eliminate hepatitis as a major public health threat by 2030 [1]. Screening for hepatitis during pregnancy is important for both the mother and the fetus; giving prophylaxis for those who are positive is widely recommended [1, 15, 16]. HIV and syphilis are routinely screened in Ethiopia. Moreover, the WHO recommended linking and integrating hepatitis services with other health services like prevention of mother-to-child transmission (PMTCT) for HIV, syphilis, and HBV integration [17]. Despite this recommendation, Ethiopia has not scaled up the HBV of PMTCT integration in the country.

In Ethiopia, there is a lack of reliable national and subnational epidemiological data on both HBV and HCV among pregnant women. However, few studies have been conducted among pregnant women with both HBV and HCV infection and coinfections. The prevalence of HBsAg and anti-HCV antibody among pregnant women varies considerably in our country from 2.4 to 8.4% and from 0.6 to 8.1%, respectively [18–21]. In Ethiopia, the coendemicity of HBV, HCV, and HIV and the same route of transmission and coinfections in pregnancy resulted in increased morbidity, mortality, and vertical transmission of these viruses from mother to child. The management of coinfected patients is very different from monoinfected patients [4, 22].

Despite the significant health burden it places on pregnant women and their infants, viral hepatitis is an ignored health problem and has given little attention in Ethiopia. Ethiopia is the least African country that has 16% institutional delivery and no birth dose HBV vaccine [9]. Antenatal care (ANC) is a key for pregnant women and a good opportunity for screening for HBV and HCV otherwise would not come. All studies in Ethiopia were done in all trimesters, but this study focused on the first trimester, which is an opportunity for early detection and intervention to reduce MTCT. All previous studies have limited geographical locations and are not representative. Since there is a scarcity of large-scale multicenter representative studies and not easy to know the burden of HBV and HCV coinfection and risk factors in the Amhara National Regional State, therefore, this study aimed to assess the magnitude of HBV and HCV infections and associated factors among first-trimester pregnant women in the Amhara National Regional State in three tertiary hospitals, Ethiopia.

## 2. Materials and Methods

### 2.1. Study Design, Area, and Period

A cross-sectional study was conducted in the Amhara National Regional State in three tertiary hospitals. The Amhara National Regional State is the second most populous region in Ethiopia, next to the Oromia region. The three hospitals are the following. The University of Gondar Comprehensive Specialized Hospital (UOGCSH) is located in Gondar, 750 km northwest of Addis Ababa. The hospital serves more than 5 million inhabitants in the Amhara region. The Felege-Hiwot Comprehensive Specialized Hospital (FHCSH) is located in Bahir Dar, the capital city of the Amhara region, and serves as a referral hospital. Debre Markos Referral Hospital (DMRH) is a zonal hospital, which serves two million people in the East Gojam Zone. The study was conducted from May 1, 2018, to September 30, 2019.

### 2.2. Study Population, Sample Size, and Sampling Technique

The study population was all pregnant women who were attending ANC in the UOGCSH, FHCSH, and DMRH during the study period. All pregnant women whose urine tests were positive for human gonadotrophic hormone (HCG) and who were in the first trimester were included in the study. Those pregnant women who had unknown gestational age and were seriously ill were excluded from the study. The sample size was calculated using a single proportion population formula based on the following assumptions: the assumption *Z* distribution with 95% confidence interval was 1.96%, the margin of error (*d*) was 2%, and proportion (*p*) has taken 50% to maximize the sample size. The final sample size was computed using the formula *n* = *Z*^2^_*α*/2_*P*(1 − *P*)/*d*^2^. After including 5% of the nonresponse rate, the final minimum sample size was 1121. A systematic random sampling technique was used to select pregnant women.

### 2.3. Data Collection

Sociodemographic and clinical characteristics of pregnant women and associated factors were collected using a pretested structured questionnaire interviewed by trained experienced senior Midwifery nurses.

### 2.4. Sample Collection, Transportation, and Processing

After getting permission from the pregnant women, 5 mL of venous blood was collected from each pregnant woman by a trained experienced senior laboratory technologist. The serum was separated within 8 h by centrifugation of whole blood at 3000 rpm for 10 min. Serum samples were separated and transferred to a nuck tube and stored at −80°C in a refrigerator until being processed using enzyme-linked immunosorbent assay (ELISA). Serum samples collected from FHCSH and DMRH were transported to the UOGCSH using cold boxes with dry ice. Hepatitis B surface antigen (HBsAg) from the serum of each pregnant woman was determined using ELISA according to the manufacturer's instructions (Linear Chemicals, S.L.U., Spain). Anti-HCV antibodies were tested using commercially available ELISA kits (DIALB Diagnostics GmbH, Vienna, Austria) according to the manufacturer's instructions. The results were read in a microplate reader at 450 nm within 30 min. In addition, the HIV test results of the pregnant women were also collected from their medical records. The HIV test was performed using rapid test kits following the national test algorithm. HIV1/2 STAT PACK was used as a screening test kit, followed by ABBON. If both test kits were discordant, they should be repeated. If both were positive, the third test (tie breaker BIOLINE) should be performed; if positive, the result was reported as positive.

### 2.5. Data Quality

The data collectors were trained on the study protocol (client approach, data collection procedures, and quality issues) prior to the start of the study. To ensure the quality of the data, questionnaires were prepared in English and translated into Amharic and retranslated back into English. The questionnaires were pretested before two weeks in the study time among 5% of pregnant women in the Poly Health Center in Gondar Town. The collected data were checked for consistency and accuracy. The standard operating procedures were strictly followed during blood sample collection, storage, and analytical process. The storage conditions and expiration date of the reagents were checked. The ELISA test was controlled by house internal known positive and negative controls processed using Wanti. Both positive and negative controls were run along with samples prepared by the manufacturer and following the manufacturer's instructions.

### 2.6. Data Analysis

Before data entry, the collected data were checked for completeness, consistency, and coded manually. The data were entered into SPSS version 20, cleaned, and recoded. Sociodemographic characteristics were analyzed using descriptive statistics such as frequency, mean, and standard deviation. Bivariate logistic regression was used to determine the association between the outcome variable and explanatory variables. All variables in the bivariate logistic regression model whose *p* value was less than or equal to 0.2 were included in the multivariable logistic regression model. The adjusted odds ratio (AOR) with a 95% confidence interval was used to check the strength of the association. Model fitness was done using Hosmer and Lemeshow statistics. Variables with a *p* value <0.05 were considered statistically significant with a 95% confidence interval.

### 2.7. Ethical Approval and Consent to Participate

Ethical approval for the study was obtained from the Institutional Review Board (IRB) of the University of Gondar. Written informed consent was obtained from each pregnant woman before enrolling in this study. The study participants' anonymity was maintained throughout the study. Study participants were identified only by their code numbers. Confidentiality of information was maintained by locking the information using a computer password. Those patients who were positive for HBsAg and anti-HCV antibody were referred for better management.

## 3. Results

### 3.1. Sociodemographic Characteristics

A total of 1121 pregnant women participated in the study. Of these, 593 (52.9%) of participants were from the University of Gondar Comprehensive Specialized Hospital and the rest were from the Bahir Dar Comprehensive Specialized Hospital and Debre Markos Referral Hospital. The mean age of study participants was 27.2 years with a standard deviation of 4.8 years. A large proportion of pregnant women 1094 (97.6%) were married. The majority of participants (895 (79.8%)) were from urban areas. Most of the study participants (1064 (94.9%)) were Orthodox Christians. More than half of the pregnant women (732 (65.3%)) were housewives. Only 20 (1.8%) pregnant women were vaccinated for HBV before the study period (Table 1).

### 3.2. Seroprevalence of HBsAg and Anti-HCV Antibody

In this study, 69 (6.2%) pregnant women had serological evidence of either infection of HBV or coinfection with HCV. The overall prevalence of HBsAg and anti-HCV was 52 (4.6%) (95% CI, 3.4–5.8%) and 18 (1.6%) (95% CI, 0.9–2.4%), respectively (Table 2). The coinfection rates of HBV and HCV were 1.4% (1/69). Ten of 52 HBV positive cases (19.2%) were coinfected with HIV. The overall HBV/HIV coinfection rate was 0.89% (10/1121). However, there were no coinfections of HCV and HIV.

The highest seroprevalence of HBsAg was 21.7% observed among unmarried pregnant women, followed by low income (9.4%) and rural residence (6.2%), respectively (Table 3). The highest seroprevalence among the associated factors was blood transfusion (7/34) (20.6%), followed by multiple sexual partners (7/61) (11.5%) (Table 4).

### 3.3. Factors Associated with HBV and HCV Infection in Pregnant Women

Unmarried pregnant women were over four times (AOR = 4.5; 95% CI: 1.9–10.6) more likely at risk of HBV infection than those who had married. Similarly, pregnant women who had HIV infection were over two times (AOR = 2.5; 95% CI: 1–5.9) more likely to develop HBV infection than their counterparts. Likewise, pregnant women with a history of blood transfusion were over seven times (AOR = 7.4; 95% CI 2.5–21.3) more likely to have HBV infection than those who did not transfuse blood. Moreover, pregnant women who had a history of sharing their toothbrush with others were 4.5 times (AOR = 4. 5, 95% CI: 1.1–18) more likely to have HBV infection than those who did not share their toothbrush (Table 4).

Pregnant women whose age was between 17 and 25 were three times (AOR = 3.1, 95% CI: 1.2–8.6) more likely to acquire HCV infection than pregnant women whose age was equal to or greater than 26 yrs. Similarly, study participants who had no educational background were five times (AOR = 5.0, 95% CI: 1.7–14.7) more likely to acquire HCV compared to study participants attending education secondary and above (Table 5).

## 4. Discussion

This is a multicenter study that examined the seroprevalence of HBsAg and anti-HCV antibody and associated factors among first-trimester pregnant women in the Amhara National Region State, Ethiopia. Our study revealed that 4.6% of pregnant women were infected with HBV. This finding is in line with the WHO intermediate classification of HBsAg prevalence, which is 2–7% [23]. Moreover, it is similar to other studies conducted previously in Bahir Dar (4.4%), Arba Minch (4.3%), Dessie (4.9%), and Egypt (5%), and South Africa (4.5%) [20, 24–27].

However, our finding is higher than the findings in studies done in the east Wolega zone (2.4%), Dawuro (3.5%), Addis Ababa (3%), Eritrea (3.2%), Turkey (2.1%), and China (3.2%) [28–33]. On the other hand, our finding is lower than the findings in studies done in Dire Dewa (8.4%), Mekele (8%), Hawassa (7.8%), Yirgalem (7.2%), Gambella (7.9%), Harar (6.9%), Tigray (5.5%), Gambia (9.2%), Tanzania (8.03%), Cameroon (6.6%), and Ghana (12.9%) [19, 21, 34–42]. There is a variation between the prevalence of our study with the studies that were conducted around the globe. The differences might be due to differences in sample size, cultural practices, socioeconomic status, availability of medical services, vaccination status, and diagnostic test kits employed in the study, and our study used ELISA confirmatory test.

The overall seroprevalence of anti-HCV antibody in the present study was found to be 1.6%. This finding is in agreement with the WHO intermediate criteria. According to the WHO classification criteria, the HCV seroprevalence graded as low is <1.5%, that graded as intermediate is 1.5%–3.5%, and that graded as high is >3.5% [12]. This finding is comparable to a study conducted in southern Ethiopia (1.8%) [43]. However, our result is higher than studies done in Tanzania (0.3%) [44] and Bahir Dar (0.6%) [20]. In contrast, our finding is lower than studies done in East Wollega (8.1%) and Egypt (6.1%) [18, 45]. On the other hand, anti-HCV antibody seroprevalence was 8.9% in Debre Markos Hospital, which is by far the highest. This finding calls for further studies in Debre Markos Hospital and around the area in the community to further explore risk factors for a high prevalence of anti-HCV antibody. Variations in seroprevalence of anti-HCV in Ethiopia and elsewhere might be due to the diagnostic test kit employed, sample size, safety precaution, awareness of transmission methods of HCV, exposure to risk factors, and geographic location. However, there was no coinfection of HCV and HIV in this study. This is in contrast to studies done in Ghana (4.1%) and southern Ethiopia (1.8%) of the coinfection of HCV/HIV investigated [36, 43]. The difference might be due to the test kit employed, awareness of transmission methods, exposure to risk factors, and geographic location.

Studies have documented that there was a coinfection of HBV and HCV among pregnant women, similar to our findings [36, 43]. This implies that HBV and HCV have the same route of transmission. In high endemic areas, coinfection of HBV and HCV is more dangerous in pregnant women and their infants than monoinfection [29, 36, 43]. In contrast to this finding, no coinfection of these viruses was detected in studies conducted in Ethiopia and elsewhere [20, 44]. The differences might be due to differences in sample sizes, awareness of transmission methods, risk factors, and diagnostic kit employed.

Of the sociodemographic characteristics, younger age and unmarried pregnant women were significantly associated with HBsAg prevalence (Table 4). This might be because, at a younger age, sexual activity is higher compared to age groups of 26 years and above. This finding agrees with other study in Gambella in Ethiopia [40]. In contrast, a study reported a high prevalence of HBsAg in pregnant women with increasing age [4]. Moreover, the probability of getting HBV infection in unmarried women is higher than that in their counterparts. This in line with a study conducted in Ethiopia [21]. This might be because unmarried pregnant women have the possibility of having more than one sexual partner whose hepatitis status is unknown compared to married pregnant women. To the contrary of this study, a high positive HBsAg is more likely in married pregnant women than in single and divorced or unmarried pregnant women [35]. The observed differences could be due to the behavior of the study participants, the sample size of the study participants, geographic location difference, and culture of the population living.

This study identified different associated risk factors for HBV infection (Table 4). Having a history of multiple sexual partners was found to be an important risk factor to acquire HBV infection. This might be because pregnant women who had multiple sexual partners were more likely to get HBV transmission through unprotected sex with different partners than those who had a single partner. This implies that there may be unsafe sexual practices within the community which is a serious practice to expose people not only to hepatitis but also to HIV, syphilis, and other sexually transmitted diseases. This finding is similar to other studies [20, 24, 39–42]. Similarly, blood transfusion has been recognized as a major risk factor for transmission of HBV [20, 33]. Having a history of blood transfusion was an independent factor significantly associated with HBV infection. This implies that blood transfusion with inadequate screening can transmit HBV. This is similar to previous studies done elsewhere [20, 21, 33]. However, the result of our study is inconsistent with other studies [39–41]. In fact, in Ethiopia, blood and blood products have been screened for HBV, HCV, HIV, syphilis, and malaria since 2001 [6]. However, in our country, screening for HBV from blood and blood products using ELISA may not detect the virus in the window period and occult hepatitis, which needs molecular techniques for accurate detection of the virus. Evidence showed that in a study done in Taiwan, among 10,727 seronegative blood donations, 12 HBV DNA was detected [46]. Therefore, it is advisable to evaluate HBsAg ELISA using molecular techniques to detect occult hepatitis.

Likewise, pregnant women with a history of tattooing on their body parts were significantly associated with HBV infection. This is in line with studies done in Addis Ababa and Arba Minch, Ethiopia [4, 24]. However, this is in contrast to other studies [38, 39]. The observed differences might be due to variations in the sample size of study participants, awareness of transmission methods of hepatitis viruses, safety precautions, traditional practices, and culture of the society.

Moreover, study participants with a family history of hepatitis infection were significantly associated with HBV infection. This might be due to study participants have a lack of awareness of transmission methods of the hepatitis virus, less precaution of sharing sharp material, traditional practices, and unsafe sexual practices. In Ethiopia, there is a belief that hepatitis disease is not transmitted from person to person; rather, it is Bat's disease or “Yewef Beshita” in the Amharic language [6]. This is similar to the study done in Tigray, Ethiopia [42]. In contrast, this is inconsistent with other studies [24, 39]. The variations might be due to differences in sample size, sharing of sharp materials, awareness of study participants regarding transmission methods, safety measures, and level of precautions.

Furthermore, pregnant women infected with HIV were over two times more likely to have HBV infection. This implies that the HBV and HIV shared routes of transmission. This finding is similar to studies done in Ethiopia and other African countries [4, 21, 35]. Viral hepatitis is a growing cause of mortality among people living with HIV. In sub-Saharan Africa (SSA), an estimated 10% of HIV-infected individuals are coinfected with HBV [47]. The coinfection rate of HBV and HIV in Ethiopia is a common phenomenon, which leads to increased morbidity and mortality than monoinfection of both viruses. HIV negatively impacts the HBV life cycle by increasing persistent HBV infection, having a higher HBV viral load, lower rates of HBeAg loss, increased cirrhosis, and increased risk of hepatocellular carcinoma [48]. Moreover, HIV/HBV coinfected pregnant women are twice as likely to test positive for HBeAg, are three times more likely to have detectable HBV DNA, and have higher HBV DNA serum concentrations as compared to those who are HBV monoinfected, thereby greatly increasing the risk of MTCT [49]. Despite the recommendation of many international and national HIV guidelines to test HBV among HIV-positive pregnant women before ART initiation, screening uptake is generally poor in Ethiopia. Therefore, screening for HBV among HIV-positive pregnant women is very essential for the mother and the child for the prevention of vertical transmission.

Interestingly, having a history of toothbrushes was an independently associated factor to acquire HBV infection. This might be due to the fact that during tooth brushing, there may be gum bleeding and HBV may be transmitted from an infected person to a susceptible person. This implies that there is low awareness of the community about the virus, the sharing of toothbrushes, and poor infection prevention practices. HBV is highly contagious and a hundred times more infectious than HIV and stays more time outside the blood than HIV. It is a tricky enveloped virus transmitted even on surfaces and acts as a nonenveloped virus in transmission [6].

In the present study, young age was significantly associated with HCV infection. This might be due to the fact that young pregnant women are sexually active and may have more sexual exposure than old pregnant women. This finding contradicts the finding of a study in Egypt which states that old age is significantly associated with HCV infection [46]. Similarly, educational status was significantly associated with HCV infection. This implies that the presence of high anti-HCV antibody prevalence in pregnant women with no educational background might be due to the lack of awareness about transmission methods, sharing sharp instruments, high prevalence of traditional practices, limited access to screening, and health care facilities.

In this study, only 20 (1.8%) pregnant women were vaccinated for HBV prevention. This percentage is less than the vaccination status of pregnant women in Gambia, 41.3% [34]. This might be due to the fact that HBV vaccination in Ethiopia started lately in 2007 [50]. This is an indication that more efforts were required to vaccinate adults born before 2007 in Ethiopia. Since 1991, the WHO recommended universal HBV vaccination of children and high-risk groups to reduce new infections and prevent progression to cirrhosis and hepatocellular carcinoma [51]. Universal vaccination is exemplary in Taiwan, together with catch-up vaccination and maternal screening, which decreases the prevalence of HBsAg from 9.8% in 1984 to 0.3% in 2009 in children younger than 15 years [52]. Likewise, in 2009, the WHO recommended a birth dose vaccine for infants within 24 h of birth [10]. However, studies have shown that birth dose vaccination may not prevent those infants born from HBeAg-positive pregnant women and have a high viral load of HBV DNA [8, 27]. Combined treatment with HBV immunoglobulin and birth dose vaccine is highly recommended and prevents 85–95% vertical transmission [51]. However, hepatitis B hyperimmune globulin is expensive and not readily available at most health institutions in Ethiopia. Thus, the authors recommended that giving birth dose vaccines within 24 h of the birth of infants is essential to prevent MTCT and integrate into HIV of PMTCT in Ethiopia. Yet, it is not routine practice to provision of birth dose vaccines in Ethiopian health institutions.

Therefore, the findings of this study will provide insights for policymakers to implement a routine practice of screening of HBV and HCV in pregnant women and immunization of infants for HBV and integration into PMTCT of HIV. However, this study has some limitations. This hospital-based study had limited generalization, but we are trying to represent the general population by increasing the sample size and multicenter and covering a large geographic area. We have not done molecular tests for HBV and HCV. The molecular DNA test is better for diagnosing occult hepatitis that has not been done in this study, which may underestimate the prevalence of HBV among pregnant women.

## 5. Conclusion and Recommendations

HBV and HCV infections are intermediate among pregnant women in the Amhara National Regional State. This finding suggests that 4.6% and 1.6% of infants born to HBV and HCV-infected pregnant women are more likely at risk of infection vertically from their mothers. We advocate that screening and integration of HBV, and HCV-infected pregnant women into PMTCT of HIV are important in Ethiopia. Factors such as being HIV-positive, history of multiple sexual partners, blood transfusion, tattooing, family history of hepatitis, and sharing of toothbrushes were significant predictors of HBV infection; similarly, age and educational status were significantly associated with HCV infection. Therefore, providing health education on HBV and HCV transmission, risk behaviors, vaccination of HBV, screening of all pregnant women, and provision of a birth dose vaccine for newly born infants within 24 hours is recommended to prevent MTCT. Moreover, further longitudinal MTCT and molecular studies will be recommended for HBV and HCV among pregnant women in Ethiopia.

## Figures and Tables

**Table 1:** Sociodemographic characteristics of pregnant women attending the Amhara national regional state in three tertiary hospitals from May 1, 2018, to September 30, 2019.

Characteristics	Category	Number	Percent
Age in year	17–25	464	39.6
	26–45	657	60.4

Marital status	Single	17	1.5
	Married	1094	97.6
	Windowed	1	0.1
	Divorced	9	0.8

Residence	Urban	895	79.8
	Rural	226	20.2

Religion	Orthodox	1064	94.9
	Muslim	49	4.4
	Protestant	7	0.6
	Catholic	1	0.1

Income (ETB)	<2000	159	14.2
	2000–4000	799	71.3
	>4000	163	14.5

Educational status	No education	254	22.7
	Read and write	6	54
	Primary	173	15.4
	High school	302	26.9
	College and above	331	29.5

Occupation	Housewife	732	65.3
	Employed	191	17
	Daily laborers	92	8.2
	Student	33	2.9
	Merchant	73	6.5

Gravida	Primigravida	409	36.5
	Multigravida	712	63.5

Parity	Nullipara	545	48.6
	Multipara	576	51.4

HBV vaccination history	Yes	20	1.8
	No	1101	98.2

Study site	UOGCSH	593	52.9
	FHCSH	447	39.9
	DMH	81	7.2

HBV=Hepatitis B virus, DMH = Debre Markos Referral Hospital, ETB = Ethiopian Birr, FHCSH = Feleg Hiwot Comprehensive Specialized Hospital, and UOGCSH = University of Gondar Comprehensive Specialized Hospital.

**Table 2:** Seroprevalence of HBsAg and anti-HCV antibody among pregnant women in the Amhara National Regional State in three tertiary hospitals from May 1, 2018, to September 30, 2019 (*n* = 1121).

Study site	Number	HBsAg status		HCV-antibody status	
		Pos *n* (%)	Neg *n* (%)	Pos *n* (%)	Neg *n* (%)
UOGCSH	593	17 (2.9)	576 (97.1)	6 (1)	587 (99)
FHCSH	443	32 (7.2)	415 (92.8)	4 (0.1)	447 (99.1)
DMRH	81	3 (3.7	78 (96.3)	8 (9.9)	73 (90.1)
Total	1121	52 (4.6%)	1069 (95.4)	18 (1.6)	1103 (98.4)

DMRH = Debre Markos Referral Hospital, FHCSH = Feleg Hiwot Comprehensive Specialized Hospital, and UOGCSH = University of Gondar Comprehensive Specialized Hospital.

**Table 3:** Binary logistic regression of sociodemographic characteristics in relation to HBV infection among pregnant women in the Amhara national regional state in three tertiary hospitals from May 1, 2018, to September 30, 2019.

Characteristics	Category	Number (%)	HBV status		Crude odds ratio (COR) (95% CI)
			Pos	Neg	
Religion	Orthodox	987 (95.0)	44 (4.5)	943 (95.5)	1
	Others	52 (5)	4 (7.7)	48 (92.3)	1.98 (0.67–5.8)
Residence	Urban	895 (79.8)	38 (4.2)	857 (95.8)	1
	Rural	226 (20.2)	14 (6.2)	212 (93.8	1.5 (0.79–2.8)
Marital status	Married	1017 (97.8)	43 (4.2)	974 (95.8)	1
	Unmarried	23 (2.2)	5 (21.7)	18 (78.3)	3.9 (1.5–9.6)^*∗*^
Age	17–25	464 (39.6)	30 (6.5)	434 (93.5)	2 (1.1–3.5)
	26–45	657 (60.4.)	22 (3.3)	635 (96.7)	1
Income	<1000	159 (14.2)	15 (9.4)	144 (90.6)	0.37 (0.2–0.7.0)
	1000–4000	799 (71.3)	30 (3.8)	769 (96.2)	1
	>4000	163 (14.5)	7 (4.3)	156 (95.7)	
Education	No education	254 (22.7)	14 (5.5)	240 (94.5)	1.77 (0.68–4.5)
	Primary + RW	234 (20.9)	11 (4.7)	223 (95.3)	1.64 (0.58–4.6)
	High school and above	633 (56.5.4)	27 (4.3)	606 (95.7)	1.07 (0.39–2.9)
Occupation	Housewife	522 (62.4)	23 (4.4)	499 (95.8)	1.49 (0.51–4.4)
	Employed	140 (16.8)	5 (3.6)	135 (96.4)	1
	Others	174 (20.8)	9 (5.2)	165 (94.8)	1.9 (0.56–6.15)
Gravida	Primigravida	409 (36.5)	24 (5.9)	385 (94.1)	1.2 (0.62–2.4)
	Multigravida	712 (63.5)	28 (3.9)	684 (961)	1
Parity	Nullipara	545 (48.6)	33 (6.1)	512 (93.9)	1.85 (0.92–3.7)
	Multipara	576 (51.4)	19 (3.5)	557 (96.7)	1

Others = Muslim, Protestant, and Catholic; RW = write and read. ^*∗∗*^ = *P* < 0.001; ^∗^ = *p* < 0.5.

**Table 4:** Bivariate and multivariate analysis of factors associated with HBV infection among pregnant women in the Amhara national regional state tertiary hospitals from May 1, 2018, to September 30, 2019.

Variable	Category	Number	HBV status		COR	Adjusted odds ratio AOR (95% CI)
			Pos	Neg		
Age	17–25	464 (39.6)	30 (6.5)	434 (93.5)	2 (1.1–3.5)	2.5 (1.3–4.5)^*∗*^
	26–45	657 (60.4)	22 (3.3)	635 (96.7)	1	
Marital status	Married	1017 (97.8)	43 (4.2)	974 (95.8)	1	
	Unmarried	23 (2.2)	5 (21.7)	18 (78.3)	4.5 (1.9–10.6)	
Tattoo	Yes	351 (31.3)	23 (6.6)	328 (93.4)	1.8 (1.0–3.1)^*∗*^	2 (1–3.8)^*∗*^
	No	770 (68.7)	29 (3.8)	741 (96.2)	1	
Dental extraction	Yes	156 (13.9)	6 (3.8)	150 (96.2)	1.3 (0.53–3)	
	No	965 (86.1)	46 (4.8)	919 (95.2)	1	
Blood transfusion	Yes	34 (3)	7 (20.6)	27 (79.4)	6 (2.5–15)^*∗∗∗*^	7.6 (2.9–16.9)^*∗∗*^
	No	1087 (97)	45 (4.1)	1042 (95.9.)	1	
Surgery	Yes	78 (8.3)	3 (3.8)	75 (96.2)	0.8 (0.24–2.7)	
	No	1043 (91.7)	49 (4.7)	994 (95.3)	1	
Sexual trans. Dis.	Yes	138 (12.3)	9 (6.5)	129 (93.5)	0.65 (0.31–1.3)	
	No	983 (87.7)	43 (4.4)	940 (95.6)	1	
HIV	Yes	113 (10.1)	10 (8.8)	103 (91.2)	2.2 (1.09–4.6)	2.5 (1–5.9)^*∗*^
	No	1008 (89.9)	42 (4.2)	966 (95.8)	1	
Abortion	Yes	203 (16)	9 (4.4)	194 (95.6)	1.1 (0.5–2.2)	
	No	918 (81.9)	43 (4.7)	875 (95.3)	1	
Unprotected sex	Yes	85 (7.5)	6 (7.1)	79 (92.9)	1.6 (0.68–4.0)	
	No	1036 (92.5)	46 (4.4)	990 (95.6)	1	
Multiple sexual partners	Yes	61 (5.4)	7 (11.5)	54 (88.5%)	3.9 (1.8–8.4)	3.2 (1.3–7.6)
	No	1060 (94.6)	45 (4.2)	1015 (95.8)	1	1
Ear pricing	Yes	248 (22.1)	18 (7.3)	230 (92.7)	1.9 (1–3.5)^*∗*^	
	No	873 (77.9)	34 (3.9)	839 (96.1)	1	
Nose piercing	Yes	50 (4.5%)	6 (12%)	44 (88%)	3 (1.2–7.5)^*∗*^	0.145
	No	1071 (95.5)	46 (4.3%)	1025 (95.7%)	1	
Diabetes	Yes	25 (2.2)	3 (12)	22 (88)	2.9 (0.84–10)	2.8 (0.7–11)
	No	1096 (98.2)	49 (4.5)	1047 (95.5)	1	
Hospitalization	Yes	144 (12.8)	6 (4.2)	138 (95.8)	0.88 (0.36–2)	
	No	977 (87.2)	46 (4.7)	931 (95.3)	1	
Family history of hepatitis	Yes	99 (8.8)	11 (11.1)	88 (88.9)	3 (1.5–6)	3.5 (1.7–7.6)^*∗∗*^
	No	981 (91.2)	41 (4)	981 (96)	1	
Sharing toothbrush	Yes	18 (1.6)	3 (16.7)	15 (83.3)	4.3 (1.2–15)	4.5 (1.1–18)^*∗*^
	No	1103 (98.4)	49 (4.4)	1054 (95.6)	1	
Sharing sharp material	Yes	62 (4.5)	3 (4.8)	59 (95.2)	1.0 (0.28–4.3)	
	No	1059 (94.5)	49 (4.6)	1010 (95.4)	1	

^*∗∗*^=*p* < 0.001; ^*∗*^=*p* < 0.05.

**Table 5:** Bivariate and multivariable analysis of factors associated with HCV infection among pregnant women in the Amhara national regional state in three tertiary hospitals from May 1, 2018, to September 30, 2019.

Variables	Category	Number	HBV status		COR (95% CI)	AOR (95% CI)
			Pos	Neg		
Age	17–25	464	11 (2.4)	453 (97.6)	2.25 (0.87–5.9)	3.2 (1.2–8.6)^*∗*^
	26–45	657	7 (1.1)	650 (98.9)	1	
Education	No education	254	9 (3.5)	245 (96.5)	3.8 (1.35–10.8)^*∗*^	5.0 (1.7–14.8)
	Primary	234	3 (1.3)	231 (98.7)	1.4 (0.33–5.5)	1.4 (0.34–5.6)
	Secondary and ab	633	6 (0.9)	627 (99.1)	1	
Tattoo	Yes	351	4 (1.1)	347 (98.9)	1.6 (0.52–4.9)	
	No	770	14 (1.8)	756 (98.2)	1	
Dental extraction	Yes	156	2 (1.3)	154 (98.7)	1.3 (0.29–5.7)	
	No	965	16 (1.7)	949 (98.3)	1	
Blood transfusion	Yes	34	0	34		
	No	1087	18 (1.7)	1069 (98.3)		
Surgery	Yes	77	2 (2.6)	75 (97.4)	0.58 (0.13–2.5)	0.50 (0.10–2.2)
	No	1044	16 (1.5)	1028 (98.5)	1	1
Sexual trans. Dis.	Yes	138	3 (2.2)	135 (97.8)	0.69 (0.19–2.4)	
	No	983	15 (1.5)	968 (98.5)	1	
HIV	Yes	113	1 (0.9)	112 (99.1)	1.9 (0.25–14.5)	
	No	1008	17 (1.7	991 (98.3)	1	
Dental extraction	Yes	156	2 (1.3)	154 (98.7)		
	No	965	16 (1.7)	900 (98.3)		
Unprotected sex	Yes	85	1 (1.3)	84 (98.7)	1.06 (0.37–3.0)	
	No	1036	17 (1.6)	1019 (98.4)	1	
Multiple sexual partners	Yes	63	2 (3.2)	61 (96.8)	0.46 (0.10–2.0)	
	No	1058	16 (1.5)	1042 (98.5)	1	
Ear pricing	Yes	248	5 (2)	243 (98)		
	No	873	13 (1.5)	860 (98.5)		
Working in hospital	Yes	20	1 (5)	19 (95)		
	No	1101	17 (1.5)	1084 (98.5)		
Diabetes	Yes	25	0	25 (100)		
	No	1096	18 (1.6)	1078 (98.4)		
Hospitalization	Yes	144	5 (3.5)	139 (96.5)	0.37 (0.13–1.06)	
	No	977	13 (1.3)	964 (98.7)	1	
Sharing sharp material	Yes	18	0	18 (100)		
	No	1103	18 (1.6)	1085 (98.4)		

^*∗∗*^=*p* < 0.001; ^*∗*^*p* < 0.05.

## Data Availability

All the data supporting the conclusion of the study are available in the paper.

## Abbreviations

ANC: Antenatal care
Anti-HCV: Anti-hepatitis C virus antibody
HBV: Hepatitis B virus
HBeAg: Hepatitis B e antigen
HBsAg: Hepatitis B surface antigen
HCV: Hepatitis C virus
DAA: Direct antiviral agents
DMRH: Debre Markos referral hospital
ELISA: Enzyme-linked immunosorbent assay
FHCSH: Felege-Hiwot comprehensive specialized hospital
HIV: Human immunodeficiency virus
MTCT: Mother-to-child transmission
PMTCT: Prevention of mother-to-child transmission
UOGCSH: University of Gondar Comprehensive Specialized Hospital
WHO: World Health Organization.

## References

[1] World Health Organization (2016). *Combating Hepatitis B and C to Reach Elimination by 2030*.

[2] Spearman C. W., Afihene M., Ally R. (2017). Hepatitis B in sub-Saharan Africa: strategies to achieve the 2030 elimination targets. *The Lancet Gastroenterology & Hepatology*.

[3] Mohd Hanafiah K., Groeger G., Flaxman A. D., Wiersma S. T. (2013). Global epidemiology of hepatitis C virus infection: new estimates of age-specific antibody to HCV seroprevalence. *Hepatology*.

[4] Desalegn Z., Wassie L., Bedimo H., Mihret A., Yehenew A. (2016). Hepatitis B and human immunodeficiency virus co-infection among pregnant women in resource-limited high endemic setting, Addis Ababa, Ethiopia: implications for prevention and control measures. *European Journal of Medical Research*.

[5] Lam N., Gotsch P. B., Langan R. C. (2010). Caring for pregnant women and newborns with hepatitis B or C. *American Family Physician*.

[6] Shiferaw F., Letebo M., Bane A. (2016). Chronic viral hepatitis: policy, regulation, and strategies for its control and elimination in Ethiopia. *BMC Public Health*.

[7] Custer B., Sullivan S. D., Hazlet T. K., Iloeje U., Veenstra D. L., Kowdley K. V. (2004). Global epidemiology of hepatitis B virus. *Journal of Clinical Gastroenterology*.

[8] Kean E., Funk A. L., Shimakawa Y. (2016). Systematic review with meta-analysis: the risk of mother to child transmission of HBV infection in sub-Sahran Africa. *Alimentary Pharmacology and Therapeutics*.

[9] Breakwell L., Tevi-Benissan C., Childs L., Mihigo R., Tohme R. (2017). The status of hepatitis B control in the African region. *The Pan African Medical Journal*.

[10] World Health Organization (2017). *Global Hepatitis Report*.

[11] Kanninen T. T., Dieterich D., Asciutti S. (2015). HCV vertical transmission in pregnancy: new horizons in the era of DAAs. *Hepatology*.

[12] World Health Organization (2017). *Hepatitis C Factsheet No 164*.

[13] Fauteux-Daniel S., Larouche A., Calderon V. (2017). Vertical transmission of hepatitis C virus: variable transmission bottleneck and evidence of midgestation in utero infection. *Journal of Virology*.

[14] Sookoian S. (2006). Liver disease during pregnancy: acute viral hepatitis. *Annals of Hepatology*.

[15] Centers for Disease Control and Prevention (2008). Recommendations for identification and public health management of persons with chronic hepatitis B virus infection. *Morbidity and Mortality Weekly Report*.

[16] National Institute for Health and Clinical Excellence (2008). *NIfHaCE: Antenatal Care: Routine Care for the Healthy Pregnant Woman*.

[17] Wang A.-L., Qiao Y.-P., Wang L.-H. (2015). Integrated prevention of mother-to-child transmission for human immunodeficiency virus, syphilis and hepatitis B virus in China. *Bulletin of the World Health Organization*.

[18] Dabsu R., Ejeta E. (2018). Seroepidemiology of Hepatitis B and C virus infections among pregnant women attending antenatal clinic in selected Health Facilities in east Wollega Zone, West Oromia, Ethiopia. *BioMed Research International*.

[19] Semaw A., Awet H., Yohannes M. (2015). Sero-prevalence of hepatitis B surface antigen and associated factors among pregnant mothers attending antenatal care service, Mekelle, Ethiopia: evidence from institutional based quantitative cross-sectional study. *World Academy of Science, Engineering and Technology: Medical and Health Sciences*.

[20] Molla S., Munshea A., Nibret E. (2015). Seroprevaence of hepatitis B surface antigen and anti HCV antibody and its associated risk factors among pregnant women attending maternity ward of Felege Hiwot Referral Hospital, Northwest Ethiopia: a cross-sectional study. *Virology Journal*.

[21] Mekonnen R., Admasu D., Belete M. (2018). Sero-Prevalence of Hepatitis B Virus and associated factors among pregnant mothers attending antenatal care in Public Health Facilities, Dire Dawa. *Journal of Medical Microbiology & Diagnosis*.

[22] World Health Organization (2011). *Management of Hepatitis B and HIV Coinfection*.

[23] Schweitzer A., Horn J., Mikolajczyk R. T., Krause G., Ott J. J. (2015). Estimations of worldwide prevalence of chronic hepatitis B virus infection: a systematic review of data published between 1965 and 2013. *The Lancet*.

[24] Yohanes T., Zerdo Z., Chufamo N. (2016). Seroprevalence and predictors of hepatitis B virus infection among pregnant women attending routine antenatal care in Arba Minch hospital, South Ethiopia. *Hepatitis Research and Treatment*.

[25] Seid M., Gelaw B., Assefa A. (2014). Sero-prevalence of HBV and HCV infections among pregnant women attending antenatal care clinic at Dessie referral hospital. *Ethiopian Journal of Health Sciences*.

[26] Kishk R., Mandour M., Elprince M. (2019). Pattern and interpretation of hepatitis B virus markers among pregnant women in North East Egypt. *Brazilian Journal of Microbiology*.

[27] Chotun N., Preiser W., van Rensburg C. J., Fernandez P., Theron G. B., Glebe D. (2017). Point-of-care screening for hepatitis B virus infection in pregnant women at an antenatal clinic: a South African experience. *PLoS One*.

[28] Sheng Q. J., Wang S. J., Wu Y. Y., Dou X. G., Ding Y. H. (2018). Hepatitis B virus serosurvey and awareness of mother-to-child transmission among pregnant women in Shenyang, China: an observational study. *Medicine*.

[29] Elsheikh R., Daak A., Elsheikh M., Karsany M., Adam I. (2007). Hepatitis B virus and hepatitis C virus in pregnant sudanese women. *Virology Journal*.

[30] Chernet A., Yesuf A., Alagaw A. (2017). Seroprevalence of Hepatitis B surface antigen and factors associated among pregnant women in Dawuro zone, SNNR, Southwest Ethiopia, across sectional study. *BMC Research Notes*.

[31] Tegegne D., Desta K., Tegbaru B., Tilahun T. (2014). Seroprevalence and transmission of hepatitis B virus among delivering women and their new born in selected health facilities, Addis Ababa, Ethiopia: a cross sectional study. *BMC Research Notes*.

[32] Fessehaye N., Berhane A., Ahmed H. (2018). Prevalence of hepatitis B virus infection and associated seromarkers among pregnant women in Eritrea. *Journal of Human Virology & Retrovirology*.

[33] Cetin S., Cetin M., Turhan E., Dolapcioglu K. (2018). Seroprevalence of hepatitis B surface antigen and associated risk factors among pregnant women. *The Journal of Infection in Developing Countries*.

[34] Bittaye M., Idoko P., Ekele B. A., Amenyi Obed S., Nyan O. (2019). Hepatitis B virus sero-prevalence amongst pregnant women in the Gambia. *BMC Infectious Diseases*.

[35] Manyahi J., Msigwa Y., Mhimbira F., Majigo M. (2017). High sero-prevalence of hepatitis B virus and human immunodeficiency virus infections among pregnant women attending antenatal clinic at Temeke municipal health facilities, Dar es Salaam, Tanzania: a cross sectional study. *BMC Pregnancy and Childbirth*.

[36] Frempong M. T., Ntiamoah P., AnnaniAkollor M. E., Owiredu W., Addai-Mensah O., Owiredu E. W. (2019). Hepatitis B and C infections in HIV-1 and non-HIV infected pregnant women in the Brong-Ahafo Region, Ghana. *PLoS ONE*.

[37] Eyong E. M., Yankam B. M., Seraphine E. (2019). The prevalence of HBsAg, knowledge and practice of hepatitis B prevention among pregnant women in the Limbe and Muyuka Health Districts of the South West region of Cameroon: a three-year retrospective study. *Pan African Medical Journal*.

[38] Metaferia Y., Dessie W., Ali I., Amsalu A. (2016). Sero-prevalence and associated risk factors of Hepatitis B virus among pregnant women in Southern Ethiopia: a hospital based cross-sectional study. *Epidemiology and Health*.

[39] Amsalu A., Ferede G., Eshetie S., Tadewos A., Assegu D. (2018). Prevalence, infectivity, and associated risk factors of hepatitis B virus among pregnant women in Yirgalem hospital, Ethiopia: implication of screening to control mother-to-child transmission. *Journal of Pregnancy*.

[40] Tunje A., Andargie M., Hiko M., Fikru C., Kejela G. (2019). Sero-prevalence of hepatitis B virus and associated factors among pregnant women in Gambella hospital, South Western Ethiopia: facility based cross-sectional study. *BMC Infectious Diseases*.

[41] Umare A., Seyoum B., Gobena T., Haile Mariyam T. (2016). Hepatitis B virus infections and associated factors among pregnant women attending antenatal care clinic at Deder hospital, Eastern Ethiopia. *PLoS One*.

[42] Mezgebo T. A., Niguse S., Gebrekidan Kahsay A., Hailekiros H., Berhe N., Asmelash T. (2018). Hepatitis B virus infection and associated risk factors among pregnant women attending antenatal care in health facilities of Tigray, Northern Ethiopia. *Journal of Medical Virology*.

[43] Bafa T. A., Egata A. D. (2020). Seroepidemiological patterns and predictors of hepatitis B, C and HIV viruses among pregnant women attending antenatal care clinic of Atat Hospital, Southern Ethiopia. *SAGE Open Medicine*.

[44] Chibwe E., Silago V., Kajoro E. (2019). Antihepatitis B surface antigen and hepatitis C antibodies among pregnant women in an urban area of Mwanza city, Tanzania. *Journal of Pregnancy*.

[45] Khamis H. H., Farghaly A. G., Shatat H. Z., El-Ghitany E. M. (2016). Prevalence of hepatitis C virus infection among pregnant women in a rural district in Egypt. *Tropical Doctor*.

[46] Li L., Chen P. J., Chen M. H., Chak K. F., Lin K. S., Tsai S. J. (2008). A pilot study for screening blood donors in Taiwan by nucleic acid amplification technology: detecting occult hepatitis B virus infections and closing the serologic window period for hepatitis C virus. *Transfusion*.

[47] Stabinski L., OʼConnor S., Barnhart M., Kahn R. J., Hamm T. E. (2015). Prevalence of HIV and hepatitis B virus co-infection in sub-Saharan Africa and the potential impact and program feasibility of hepatitis B surface antigen screening in resource-limited settings. *Journal of Acquired Immune Deficiency Syndromes*.

[48] Thio C. L. (2009). Hepatitis B and human immunodeficiency virus coinfection. *Hepatology*.

[49] Kourtis A. P., Bulterys M., Hu D. J., Jamieson D. J. (2012). HIV–HBV co-infection—a global challenge. *The New England Journal of Medicine*.

[50] Fedral Ministry of Health (2010). *Ethiopia: National Expanded Programme on Immunization Comprehensive Multi-Year Plan 2011–2015*.

[51] Cassidy A., Mossman S., Olivieri A., Ridder M. D., Leroux-Roels G. (2011). Hepatitis b vaccine effectiveness in the face of global HBV genotype diversity. *Expert Review of Vaccines*.

[52] Chan C. Y., Lo K. J., Lee S. D. (2004). Legend of hepatitis B vaccination: the Taiwan experience literature review. *Journal of Gasteroenterology and Hepatology*.

